# Duration of aromatase inhibitor use and long-term cardiovascular risk in breast cancer survivors

**DOI:** 10.1093/jncics/pkaf009

**Published:** 2025-01-28

**Authors:** Yuhan Huang, Marilyn L Kwan, Susan R Heckbert, Nicholas L Smith, Megan Othus, Cecile A Laurent, Janise M Roh, Eileen Rillamas-Sun, Valerie S Lee, Tatjana Kolevska, Richard K Cheng, Carlos Irribarren, Mai Nguyen-Huynh, Dawn L Hershman, Lawrence H Kushi, Heather Greenlee

**Affiliations:** Department of Epidemiology, School of Public Health, University of Washington, Seattle, WA, United States; Division of Public Health Sciences, Fred Hutchinson Cancer Center, Seattle, WA, United States; Division of Research, Kaiser Permanente Northern California, Pleasanton, CA, United States; Department of Epidemiology, School of Public Health, University of Washington, Seattle, WA, United States; Department of Epidemiology, School of Public Health, University of Washington, Seattle, WA, United States; Kaiser Permanente Washington Health Research Institute, Kaiser Permanente Washington, Seattle, WA, United States; Department of Veterans Affairs Office of Research and Development, Seattle Epidemiologic Research and Information Center, Seattle, WA, United States; Department of Epidemiology, School of Public Health, University of Washington, Seattle, WA, United States; Division of Public Health Sciences, Fred Hutchinson Cancer Center, Seattle, WA, United States; Division of Research, Kaiser Permanente Northern California, Pleasanton, CA, United States; Division of Research, Kaiser Permanente Northern California, Pleasanton, CA, United States; Division of Public Health Sciences, Fred Hutchinson Cancer Center, Seattle, WA, United States; Division of Research, Kaiser Permanente Northern California, Pleasanton, CA, United States; Department of Oncology, Kaiser Permanente Vallejo Medical Center, Vallejo, CA, United States; Division of Cardiology, University of Washington School of Medicine, Seattle, WA, United States; Division of Research, Kaiser Permanente Northern California, Pleasanton, CA, United States; Division of Research, Kaiser Permanente Northern California, Pleasanton, CA, United States; Department of Neurology, Kaiser Permanente Walnut Creek Medical Center, Walnut Creek, CA, United States; Department of Medicine, Columbia University Irving Medical Center, New York, NY, United States; Division of Research, Kaiser Permanente Northern California, Pleasanton, CA, United States; Division of Public Health Sciences, Fred Hutchinson Cancer Center, Seattle, WA, United States; Division of Research, Kaiser Permanente Northern California, Pleasanton, CA, United States; Division of Cardiology, University of Washington School of Medicine, Seattle, WA, United States

## Abstract

**Background:**

There are limited data on duration of aromatase inhibitor (AI) and cardiovascular disease (CVD) risk in breast cancer (BC) survivors. We examined the risk of CVD and mortality associated with the duration of AI use in postmenopausal women with early stage hormone receptor-positive BC.

**Methods:**

Postmenopausal women diagnosed with hormone receptor-positive BC (*n* = 5853) who used an AI were included. Cause-specific hazards models estimated hazard ratios (HRs) and 95% confidence intervals (CIs) for associations between AI use duration (short term: >0 and <2 years; intermediate term: ≥2 and <5 years; long term: ≥5 years) and CVD and mortality outcomes. The landmark method was used to avoid immortal time bias; the selected landmark was 6 years after BC diagnosis.

**Results:**

Anastrozole was the AI predominantly prescribed (95.4%). Over a median follow-up of 3 years for women who survived 6 years after BC diagnosis, a lower risk of stroke was observed in intermediate-term AI users (HR = 0.60, 95% CI = 0.37 to 0.96) and long-term AI users (HR = 0.51, 95% CI = 0.30 to 0.85), than in short-term AI users. The longer duration of AI use was also associated with lower risk of all-cause mortality and non-CVD-related mortality. In addition, long-term AI users were at 37% lower risk of CVD-related mortality than short-term AI users. No statistically significant differences were observed in risks of major adverse cardiovascular events, ischemic heart disease, and heart failure across the 3 groups.

**Conclusion:**

Among postmenopausal women with early stage hormone receptor-positive BC who survived 6 years after BC diagnosis, longer duration of AI use was not associated with elevated CVD risk.

## Introduction

Over 4 million women with a history of breast cancer (BC) live in the United States, with approximately 310 720 new patients expected in 2024.^1^ About 80% of BC patients are hormone receptor-positive,^2^ and estrogen can promote the growth and proliferation of these BC cells. Although endogenous estrogen is detrimental to women with hormone receptor-positive BC, it may protect cardiovascular health through direct and indirect effects such as promoting vasorelaxation of vascular endothelial cells and reducing oxidative stress.^3-5^

Aromatase inhibitors (AIs) are used to halt the production of endogenous estrogen and treat hormone receptor-positive BC in postmenopausal women. Aromatase inhibitor use may increase cardiovascular disease (CVD) risk because it decreases the levels of potentially cardioprotective endogenous estrogen. Previous studies have yielded conflicting results and have focused on CVD risk comparing AI use to another endocrine therapy drug, tamoxifen, or nonusers of endocrine therapy.^6-14^ The inconsistency of these results may stem from the heterogeneous characteristics of study populations, various definitions of endocrine therapy use and CVD outcomes, and different approaches in calculating person-time. Aromatase inhibitors are usually prescribed for 5 years. However, patients may discontinue AI treatment early due to side effects and cost. Despite BC guidelines and results from clinical trials emphasizing the importance of long-term AI use (≥5 years) given its favorable clinical outcomes,^15-18^ few studies have examined the duration of AI exposure when investigating AI-related cardiovascular disease risk.

We previously found that ever use of AIs in postmenopausal women was not associated with elevated CVD risk compared with nonusers of endocrine therapy.^19^ However, duration of AI use may affect cardiovascular health as well. Thus, we conducted a prospective cohort study in postmenopausal women with early stage hormone receptor-positive BC to examine the associations of duration of AI use with CVD risk and mortality.

## Methods

### Study population

**Figure 1.**
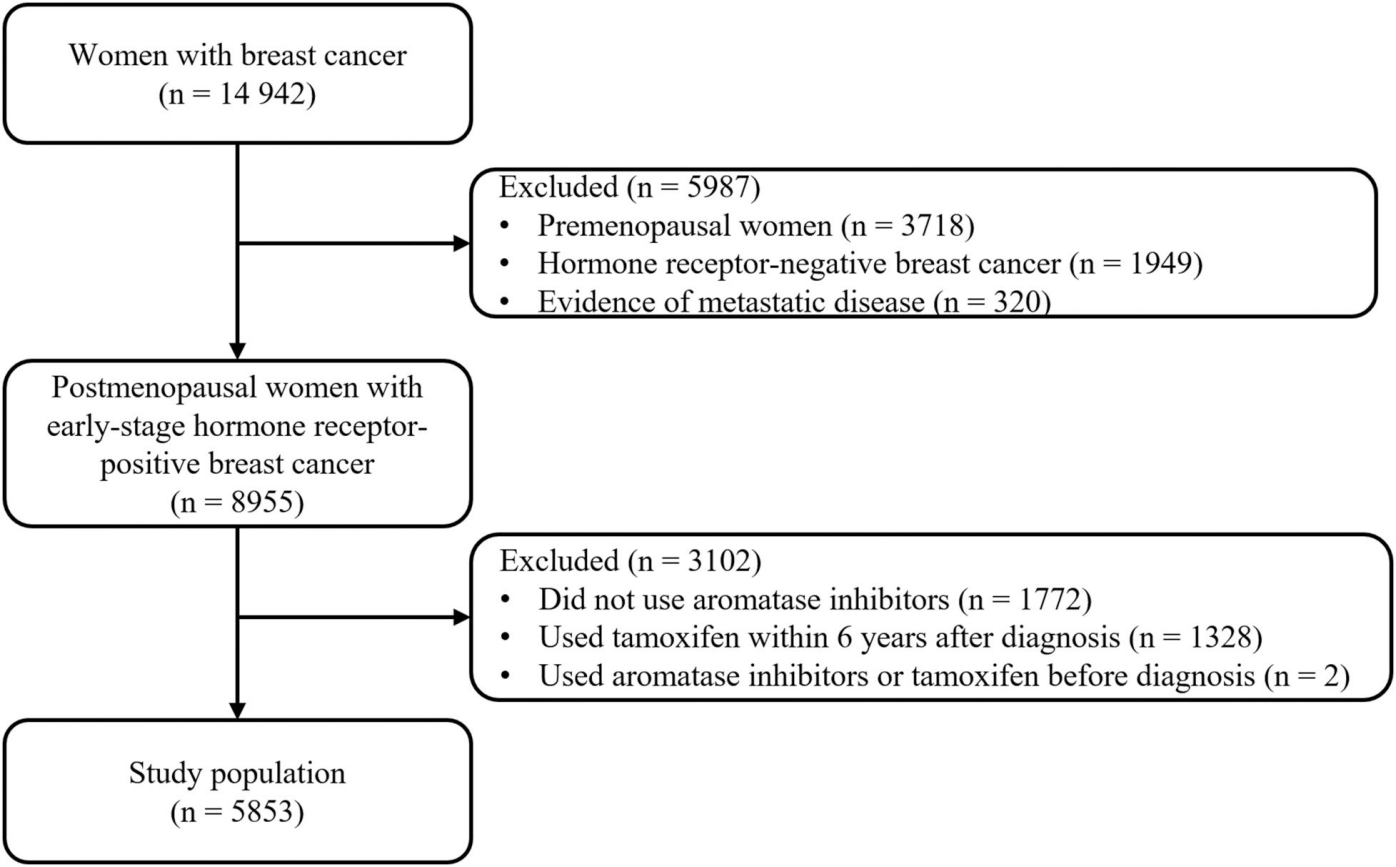
The strengthening the reporting of observational studies in epidemiology diagram of the study population.

The Pathways Heart Study (R01CA214057, multiple principal investigators (MPIs): M.L.K. and H.G.) uses Kaiser Permanente Northern California (KPNC) electronic health record (EHR) data to examine incident CVD and cardiometabolic risk factors in women who were diagnosed with BC from November 2005 to March 2013.^20^ Stages I-IV BC patients who were female aged 21 years or older without a history of any invasive cancer were included in the Pathways Heart Study.^20^ This was a data-only study that used existing protected health information from the EHRs of KPNC and is therefore, regulated by the Health Insurance Portability and Accountability Act (HIPAA) Privacy Rule. The inclusion criteria for this analysis were (1) postmenopausal women with a first-ever diagnosis of stages I-III BC; (2) women who were eligible for endocrine therapy (with estrogen or progesterone receptor-positive BC); and (3) women who had at least 1 pharmacy fill of an AI. We excluded: (1) women with evidence of metastatic disease (*n* = 320)^21^^,^^22^; (2) women with AI and/or tamoxifen prescriptions prior to their BC diagnosis (*n* = 2); (3) women who had ever been treated with tamoxifen within 6 years after BC diagnosis (*n* = 1772). Figure 1 presents the strengthening the reporting of observational studies in epidemiology (STROBE) diagram of the study population.

### Data collection and measures

Covariate data were obtained from KPNC EHR, including age, race, socioeconomic characteristics (ie, percent neighborhood education of college graduate or higher and neighborhood median household income), body mass index (BMI), smoking history, and the Comorbidity Point Score Version 2 (COPS2) at baseline, which was defined as the date of BC diagnosis. Comorbidity Point Score Version 2 is a measure of comorbidity developed and used internally at KPNC.^23^ It is calculated using diagnostic codes of inpatient and outpatient encounters occurring in the year before diagnosis.^23^ Age greater or equal to 50 years was used as a proxy for postmenopausal status, with a subset who self-reported their status if they participated in the main Pathways Study.^24^ Baseline comorbidities including diabetes, dyslipidemia, and hypertension were defined using registry data, International Classification of Diseases (ICD) diagnostic codes, medication use, and/or laboratory results.^20^ Baseline CVD history was defined using ICD diagnostic codes and Current Procedural Terminology (CPT) codes from inpatient, ambulatory, and emergency department encounters and/or hospital discharge records within 3 years prior to the BC diagnosis.^20^ Data on cancer characteristics, chemotherapy, radiation therapy, and endocrine therapy were obtained from the KPNC Cancer Registry and supplemented by procedure, infusion, and outpatient pharmacy data sources. Total duration of AI use was calculated by summing the total days supplied starting from the earliest dispensing date after BC diagnosis and ending at 6 years after BC diagnosis (ie, the landmark time). Because BC guidelines recommend women with hormone receptor-positive BC use endocrine therapy for at least 5 years and many discontinue the treatment early,^15^^,^^18^ participants were classified into 1 of 3 groups: (1) short-term AI use (>0 and <2 years); (2) intermediate-term AI use (≥2 and <5 years); (3) long-term AI use (≥5 years).

### Outcome ascertainment

The KPNC EHR was used to identify cardiovascular events through December 31, 2021, according to ICD diagnostic codes and CPT codes from inpatient, ambulatory, and emergency department encounters and/or hospital discharge records.^20^ Data on death date and cause of death through December 31, 2021, were obtained from linkage to the KPNC mortality file, which contains data from the California State Department of Vital Statistics, US Social Security Administration, National Death Index, and Kaiser Permanente membership and utilization data. The primary CVD outcome was a composite outcome of major adverse cardiovascular events (MACE), including ischemic heart disease (IHD), heart failure (HF), stroke, and CVD-related mortality. Cardiovascular disease-related mortality was defined as death due to IHD, stroke, HF, cardiomyopathy, arrhythmia, cardiac arrest, myocarditis/pericarditis, valvular disease, venous thromboembolism, and carotid disease. Secondary CVD outcomes included IHD, HF, and stroke. Fatal CVD events were included in the corresponding CVD outcomes. Mortality outcomes included all-cause mortality, CVD-related mortality, and non-CVD-related mortality.

### Statistical analysis

The mean and SD for continuous variables and frequency and percentage for categorical variables were used to summarize the baseline characteristics of study participants. To avoid immortal time bias, the landmark method was used to calculate person time.^25^ The landmark method refers to the analysis designating a time point occurring during the follow-up period (known as the landmark time) and analyzing only those patients who have not developed outcome events by the landmark time. Because over 90% of users initiated endocrine therapy within 1 year after BC diagnosis and long-term AI use was defined as greater or equal to 5 years, most participants had to survive the first 6 years since BC diagnosis to become long-term AI users. Therefore, this study used 6 years after BC diagnosis as the landmark time. Participants who were treated with tamoxifen or developed outcome events before the landmark time were excluded. Follow-up commenced 6 years after BC diagnosis and ended on the date the outcome event occurred, health plan disenrollment, death, or end of study (December 31, 2021), whichever occurred first. Cause-specific hazards models were used to account for competing risks and to estimate hazard ratios (HRs) and 95% confidence intervals (CIs) of the association of duration of AI use with CVD and mortality outcomes. Complete case analyses were performed. These models were adjusted for potential confounders including age at BC diagnosis, race, percent neighborhood education of college graduate or higher, neighborhood median household income, BMI, history of ever smoking, American Joint Committee on Cancer (AJCC) stage, chemotherapy, and radiation, and additionally adjusted for precision variables including diabetes, dyslipidemia, hypertension, COPS2, and CVD history. All analyses were performed using R (version 4.3.2, R development Core Team 2023).^26^ Statistical tests were 2-sided, and a *P* value less than 0.05 was considered to be statistically significant.

## Results

### Baseline characteristics

A total of 5853 postmenopausal women who were diagnosed with early stage hormone receptor-positive BC from 2005 to 2013 and used AIs within 6 years after diagnosis were included in this study. The average age of study participants was 66.2 (SD = 8.9) years old and 70% were White (Table 1). Anastrozole was the most commonly prescribed AI agent (*n* = 5582, 95.4%). Some women switched from 1 AI agent to another due to intolerance and thus the sum of percentage of AI type exceeded 100%. Compared with short-term AI users, intermediate-term AI users were more likely to be younger, have an obese BMI, receive chemotherapy, receive radiation, have a lower COPS2 (ie, fewer comorbidities), and were less likely to have prevalent CVD and hypertension at baseline. Compared with short-term AI users, long-term AI users were more likely to be younger, live in neighborhoods with higher median household income, receive chemotherapy, receive radiation, have a lower COPS2 (ie, fewer comorbidities), and were less likely to have prevalent CVD at baseline.

**Table 1.** Characteristics of study participants at breast cancer diagnosis by the duration of aromatase inhibitor use (*n* = 5853).

	Short term (0-2 y) (*n* = 1159)	Intermediate term (2-5 y) (*n* = 2853)	Long term (≥5 y) (*n* = 1841)	Overall (*n* = 5853)
**Age at diagnosis, mean (SD), y**	68.4 (10.4)	66.0 (8.8)	65.1 (7.7)	66.2 (8.9)
**Race/ethnicity, *n* (%)**				
American Indian and Alaska Native	8 (0.7%)	17 (0.6%)	10 (0.5%)	35 (0.6%)
Asian	114 (9.8%)	332 (11.6%)	306 (16.6%)	752 (12.8%)
Black	83 (7.2%)	168 (5.9%)	91 (4.9%)	342 (5.8%)
Hispanic	127 (11.0%)	323 (11.3%)	155 (8.4%)	605 (10.3%)
Pacific Islander	2 (0.2%)	11 (0.4%)	6 (0.3%)	19 (0.3%)
White	825 (71.2%)	2002 (70.2%)	1273 (69.1%)	4100 (70.0%)
**Percentage of neighborhood education of college graduate or higher, mean (SD), %**				
Mean (SD)	22.9% (11.7%)	23.5% (11.5%)	24.3% (11.1%)	23.6% (11.4%)
Missing	12 (1.0%)	32 (1.1%)	14 (0.8%)	58 (1.0%)
**Neighborhood median household income, mean (SD), $**				
Mean (SD)	78 600 (34 800)	80 800 (33 600)	85 100 (34 800)	81 700 (34 300)
Missing	12 (1.0%)	32 (1.1%)	14 (0.8%)	58 (1.0%)
**BMI category, *n* (%)**				
Underweight	8 (0.7%)	26 (0.9%)	11 (0.6%)	45 (0.8%)
Normal	341 (29.4%)	732 (25.7%)	514 (27.9%)	1587 (27.1%)
Overweight	381 (32.9%)	898 (31.5%)	615 (33.4%)	1894 (32.4%)
Obese	428 (36.9%)	1197 (42.0%)	701 (38.1%)	2326 (39.7%)
Missing	1 (0.1%)	0 (0%)	0 (0%)	1 (0.0%)
**BMI, kg/m^2^**				
Mean (SD)	28.9 (6.48)	29.5 (6.50)	29.1 (6.37)	29.2 (6.46)
Missing	1 (0.1%)	0 (0%)	0 (0%)	1 (0.0%)
**History of smoking, *n* (%)**				
Never	559 (48.2%)	1455 (51.0%)	1032 (56.1%)	3046 (52.0%)
Ever	497 (42.9%)	1201 (42.1%)	691 (37.5%)	2389 (40.8%)
Missing	103 (8.9%)	197 (6.9%)	118 (6.4%)	418 (7.1%)
**AJCC stage, *n* (%)**				
Stage I	694 (59.9%)	1621 (56.8%)	1064 (57.8%)	3379 (57.7%)
Stage II	361 (31.1%)	985 (34.5%)	601 (32.6%)	1,947 (33.3%)
Stage III	104 (9.0%)	247 (8.7%)	176 (9.6%)	527 (9.0%)
**AI type, *n* (%)**				
Anastrozole	1092 (94.2%)	2725 (95.5%)	1765 (95.9%)	5582 (95.4%)
Letrozole	168 (14.5%)	380 (13.3%)	251 (13.6%)	799 (13.7%)
Exemestane	93 (8.0%)	232 (8.1%)	120 (7.0%)	454 (7.8%)
**Received chemotherapy, *n* (%)**				
No	926 (79.9%)	1970 (69.1%)	1172 (63.7%)	4068 (69.5%)
Yes	228 (19.7%)	870 (30.5%)	662 (36.0%)	1760 (30.1%)
Missing	5 (0.4%)	13 (0.5%)	7 (0.4%)	25 (0.4%)
**Received radiation, *n* (%)**				
No	651 (56.2%)	1419 (49.7%)	924 (50.2%)	2994 (51.2%)
Yes	497 (42.9%)	1405 (49.2%)	906 (49.2%)	2808 (48.0%)
Missing	11 (0.9%)	29 (1.0%)	11 (0.6%)	51 (0.9%)
**COPS2**				
Mean (SD)	18.8 (22.8)	13.9 (13.6)	13.0 (11.5)	14.6 (15.5)
Missing	11 (0.9%)	29 (1.0%)	11 (0.6%)	51 (0.9%)
**History of prior CVD, Yes, *n* (%)**	176 (15.2%)	293 (10.3%)	165 (9.0%)	634 (10.8%)
**History of diabetes, Yes, *n* (%)**	307 (26.5%)	765 (26.8%)	482 (26.2%)	1554 (26.6%)
**History of dyslipidemia, Yes, *n* (%)**	755 (65.1%)	1974 (69.2%)	1308 (71.0%)	4037 (69.0%)
**History of hypertension, Yes, *n* (%)**	794 (68.5%)	1995 (69.9%)	1343 (72.9%)	4132 (70.6%)

Abbreviations: AI = aromatase inhibitor; AJCC = American Joint Committee on Cancer; BMI = body mass index; COPS2 = Comorbidity Point Score Version 2; CVD = cardiovascular disease.

Definition of BMI category: underweight: BMI less than 18.5 kg/m^2^; normal: BMI 18.5 to <25 kg/m^2^; overweight: BMI 25.0 to <30 kg/m^2^; obese: BMI 30 or higher kg/m^2^.

Endocrine therapy was defined at the landmark time, ie, 6 years after breast cancer diagnosis.

### CVD risk and mortality risk

Table 2 presents the results of the main association analysis. Over a median follow-up of 3.0 (interquartile range = 0.2-5.2) years with follow-up time starting from 6 years after BC diagnosis, intermediate-term and long-term AI users were less likely to develop stroke (HR = 0.60, 95% CI = 0.37 to 0.96 and HR = 0.51, 95% CI = 0.30 to 0.85, respectively) than short-term AI users. Inverse, yet nonsignificant, associations between longer duration of AI use and risk of MACE, IHD, and HF were observed.

**Table 2.** The associations of the duration of AI use with CVD and mortality outcomes using six years after breast cancer diagnosis as the landmark.

Outcome	Duration of AI use	No. of events	No. of excluded events	No. of participants	HR (95% CI)
CVD outcomes					
Ischemic heart disease	Short term (0-2 y)	22	68	974	Ref
	Intermediate term (2-5 y)	85	161	2453	0.82 (0.51 to 1.33)
	Long term (≥5 y)	59	73	1629	0.68 (0.41 to 1.13)
Stroke	Short term (0-2 y)	25	41	1001	Ref
	Intermediate term (2-5 y)	55	62	2552	0.60 (0.37 to 0.96)
	Long term (≥5 y)	35	14	1688	0.51 (0.30 to 0.85)
Heart failure	Short term (0-2 y)	32	90	952	Ref
	Intermediate term (2-5 y)	110	146	2468	0.87 (0.58 to 1.30)
	Long term (≥5 y)	89	60	1642	0.94 (0.62 to 1.42)
MACE	Short term (0-2 y)	67	193	849	Ref
	Intermediate term (2-5 y)	227	340	2274	0.84 (0.63 to 1.10)
	Long term (≥5 y)	162	133	1569	0.75 (0.56 to 1.01)
Mortality outcomes					
All death	Short term (0-2 y)	177	307	735	Ref
	Intermediate term (2-5 y)	442	302	2312	0.66 (0.55 to 0.79)
	Long term (≥5 y)	273	16	1686	0.56 (0.46 to 0.68)
CVD death	Short term (0-2 y)	40	60	982	Ref
	Intermediate term (2-5 y)	107	75	2539	0.75 (0.52 to 1.08)
	Long term (≥5 y)	65	6	1696	0.63 (0.42 to 0.95)
Non-CVD death	Short term (0-2 y)	137	247	795	Ref
	Intermediate term (2-5 y)	335	227	2387	0.63 (0.51 to 0.77)
	Long term (≥5 y)	208	10	1692	0.53 (0.42 to 0.66)

Abbreviations: AI = aromatase inhibitor; CVD = cardiovascular disease; HR = hazard ratio; IHD = ischemic heart disease; MACE = major cardiovascular adverse events.

HRs adjusted for age at breast cancer diagnosis (continuous), race (White, non-White), percent neighborhood education of college graduate or higher (continuous), neighborhood median house income (continuous), body mass index (continuous), ever smoking (yes, no), American Joint Committee on Cancer stage (I, II, III), chemotherapy (yes, no), radiation (yes, no), diabetes (yes, no), dyslipidemia (yes, no), hypertension (yes, no), Comorbidity Point Score Version 2 (continuous), CVD history (yes, no).

No. of excluded events refers to women who developed the outcome event before the landmark time.

MACE: a composite outcome of IHD, stroke, heart failure, and CVD death.

CVD death: death due to IHD, stroke, heart failure, cardiomyopathy, arrhythmia, venous thromboembolic disease, cardiac arrest, myocarditis/pericarditis, valvular disease, and carotid disease.

Intermediate-term and long-term AI users had lower risk of all-cause mortality and non-CVD-related mortality as compared with short-term AI users. In addition, long-term AI users had a 37% lower risk of CVD-related mortality (HR = 0.63, 95% CI = 0.42 to 0.95), compared with short-term AI users.

## Discussion

In this prospective cohort study of postmenopausal women who used AIs to treat early stage hormone receptor-positive BC, longer duration of AI use was associated with lower risks of stroke, all-cause mortality, CVD-related mortality, and non-CVD-related mortality. The risk of MACE, IHD, and HF was not associated with intermediate-term and long-term AI users compared with short-term AI users. The results of our study need to be interpreted in relation to the selected landmark time; our study can only examine CVD and mortality risk comparing women who use AIs for 2-5 years or 5 years and more to those who used AIs for less than 2 years within 6 years after BC diagnosis.

Our finding of 40%-50% reduction in risk of stroke with longer duration of AI use needs to be interpreted with caution, as it was derived in a small sample size and was unexpected as our hypothesis was AIs might increase CVD risk. Although the biological mechanism of the observed association is unclear, one hypothesis is that AI users often take bisphosphonates to prevent bone fractures, and bisphosphonates may also be cardioprotective. However, evidence of the cardioprotective effect of bisphosphonates is mixed.^27-29^ Other possible explanations include confounding by unmeasured factors such as diet and alcohol intake, residual confounding by inadequate adjustment, and bias by the healthy adherer effect, which arises when healthy behaviors are associated with both the adherence to treatment and the outcome of interest.^30^ In our study, intermediate- and long-term AI users were more likely to have prior history of CVD and higher comorbidity score, compared with short-term AI users, indicating that they were less healthy than short-term AI users. Although the direction of bias caused by unmeasured and residual confounding is unpredictable, when the healthy adherer effect exists, longer term AI use will appear more beneficial than shorter term AI use. However, it is also possible that our hypothesis was wrong, and AI use does not elevate CVD risk, which is consistent with our previous findings.^19^

We previously reported that BC survivors who used AIs were at higher CVD risk than women without a history of BC,^31^ but this study did not find elevated CVD risk associated with AI use. The results of our previous study may be biased due to confounding by indication; BC and CVD have some common risk factors and BC survivors who received endocrine therapy are sicker than women without a history of BC, and thus AI use would appear to be harmful when comparing BC survivors receiving AI treatment to women without a history of BC.

To our knowledge, this study was the first study to investigate CVD and mortality risk long-term (≥5 years) and intermediate-term (2-5 years) AI use compared with short-term (less than 2 years) AI use. Previous studies comparing short-term AI use to nonusers of endocrine therapy regarding CVD risk had conflicting results.^8^^,^^13^ Haque et al. conducted a retrospective cohort study to examine the association between duration of AI use and CVD risk in postmenopausal women with hormone receptor-positive BC and without prior CVD.^13^ They started follow-up at BC diagnosis and defined duration of AI use by summing the total days supplied starting from the earliest dispensing date after BC diagnosis and ending at one of the study end points, making the study subject to immortal time bias.^13^ They reported that using AI for more than 3 years was associated with decreased risk of HF/cardiomyopathy (HR = 0.56, 95% CI: 0.38 to 0.83), while using AI for 1 year was associated with increased risks of HF/cardiomyopathy (HR = 1.29, 95% CI = 1.01 to 1.64) and stroke (HR = 1.41, 95% CI = 1.94 to 1.92), when compared with nonusers of endocrine therapy.^13^ They did not observe difference in risks of cardiac ischemia, stroke, and HF/cardiomyopathy comparing AI use for 2-3 years with nonuse of endocrine therapy.^13^ Another retrospective cohort study conducted by Sund et al. also examined the association between the duration of AI use and CVD risk.^8^ They started follow-up at 6 months after BC diagnosis and did not report the method to calculate duration of AI use.^8^ They reported an elevated risk of acute IHD (HR = 2.03, 95% CI = 1.15 to 3.58) comparing AI use for more than 4 years with nonuse of endocrine therapy or AI use less than 1 year in postmenopausal women with BC.^8^ No associations of the duration of AI use with HF/cardiomyopathy and stroke were observed.^8^

Our study has several strengths. First, we followed study participants for a long period. The median follow-up time starting from BC diagnosis was 9.0 (interquartile range = 6.2-11.2) years. Thus, we were able to estimate CVD and mortality risk related to long-term AI use (≥5 years). Second, our study accounted for immortal time bias using the landmark method. Women who used AIs for 5 years or more had to survive long enough (ie, 6 years after BC diagnosis in our study) without developing the outcome events. When they were compared with women who only used AIs for 2 years, there is a time interval during which long-term AI users cannot develop the outcome of interest and thus immortal time bias would be introduced if not utilizing appropriate statistical methods, as suggested by our sensitivity analyses. In addition, this study adopted short-term AI use rather than nonuse of endocrine therapy as the reference group in regression models to avoid confounding by indication, which occurs when clinical indication for treatment also affects the outcome of interest. We also excluded participants who were treated by tamoxifen to avoid bias by drug switching.

Our study also has several limitations. First, the precision of our estimates was limited because we used 6 years after BC diagnosis as the landmark and excluded participants who developed the outcome before the landmark time. However, the selected landmark is necessary because a shorter landmark time will provide an incomplete capture of long-term AI use. Second, our study did not consider different formulations of AIs. However, results from randomized clinical trials suggest no significant difference in CVD risk in the 3 AIs (ie, anastrozole, letrozole, and exemestane).^32-36^ Furthermore, we enrolled women diagnosed with BC from 2005 to 2013, and their utilization patterns of endocrine therapy may be different from what it is being used now. Additionally, we did not examine the association between the duration of AI use and severe CVD events, such as myocardial infarction, HF hospitalization, and revascularization, although our definition of IHD included acute myocardial infarction and revascularization and our definition of HF included HF hospitalization. These severe CVD events are closely related to CVD-related mortality, and assessing the risk of these specific CVD events related to longer term AI exposure is also important for understanding the relationship between AI use and CVD-related mortality. Thus, we cannot rule out the possibility that our observed inverse association between longer term AI use and CVD-related mortality is due to low incidence of these severe CVD events. Last, we did not account for downstream effects of AIs on triglycerides and cholesterol levels. However, our previous work found that BC survivors receiving any endocrine therapy were at a similar risk of dyslipidemia compared with participants without BC,^20^ suggesting that the influence of AI use on lipid levels appears to have limited clinical significance.

In postmenopausal women who used AIs to treat early stage hormone receptor-positive BC, we found evidence that longer duration of AI use assessed at 6 years after BC diagnosis was associated with lower risk of stroke, all-cause mortality, CVD-related mortality, and non-CVD-related mortality. Our findings suggest long-term AI use might be cardiovascular safe in postmenopausal women with early stage hormone receptor-positive BC. Future studies with large sample size and addressing healthy adherer effect are needed to examine the association between long-term AI use and CVD risk.

## Data Availability

Data cannot be shared publicly because public availability would compromise patient privacy by revealing potentially identifiable information (eg, dates of diagnoses) and Kaiser Permanente Northern California privacy regulations. Data are available from the Kaiser Permanente Northern California Institutional Review Board and Pathways Study Data Access Committee (contact via DOR-Pathways@kp.org) for researchers who meet the criteria for access to confidential data.
